# High *TRGV 9* Subfamily Expression Marks an Improved Overall Survival in Patients With Acute Myeloid Leukemia

**DOI:** 10.3389/fimmu.2022.823352

**Published:** 2022-02-10

**Authors:** Xueting Kong, Jiamian Zheng, Xiaxin Liu, Wandi Wang, Xuan Jiang, Jie Chen, Jing Lai, Zhenyi Jin, Xiuli Wu

**Affiliations:** ^1^Institute of Hematology, School of Medicine, Jinan University, Guangzhou, China; ^2^Department of Hematology, First Affiliated Hospital, Jinan University, Guangzhou, China; ^3^Department of Pathology, School of Medicine, Jinan University, Guangzhou, China

**Keywords:** acute myeloid leukemia, γδ T cells, *TRGV* repertoire, clonality, prognosis

## Abstract

**Background:**

Heterogeneous T cells in acute myeloid leukemia (AML) have the combinatorial variety generated by different T cell receptors (TCRs). γδ T cells are a distinct subgroup of T cells containing TCRγ (*TRGV*) and TCRδ (*TRDV*) subfamilies with diverse structural and functional heterogeneity. Our previous study showed that clonally expanded *TRDV* T cells might benefit the immune response directed against AML. However, the features of the *TRGV* repertoire in AML remain unknown. To fully characterize the features of γδ T cells, we analyzed the distribution and clonality of *TRGV I-III* subfamilies (*TRGV II* is also termed *TRVG 9*), the proportions of γδ T cell subsets, and their effects on the overall survival (OS) of patients with AML.

**Methods:**

In this study, the complementarity-determining region 3 (CDR3) size of *TRGV* subfamilies in γδ T cells of peripheral blood (PB) from *de novo* AML patients were analyzed by Genescan analysis. Expression levels of *TRGV* subfamilies were performed by real-time quantitative PCR. The proportions of total γδ T cells and their Vγ9^+^ Vδ2^+^ T cells subsets were detected by multicolor flow cytometry assay. We further compared the correlation among the *TRGV* gene expression levels, the proportion of Vγ9^+^ Vδ2^+^ T cells, and OS in AML.

**Results:**

We first found that the distribution pattern and clonality of *TRGV* subfamilies were changed. The expression frequencies and gene expression levels of three *TRGV* subfamilies in AML samples were significantly lower than those in healthy individuals (HIs). Compared with HIs, the proportions of total γδ T cells and Vγ9^+^ Vδ2^+^ T cells were also significantly decreased in patients with AML. In addition, patients with AML who had higher expression levels of the *TRGV* gene and higher proportion of Vγ9^+^ Vδ2^+^ T cells showed better OS than their counterparts. Furthermore, high expression levels of *TRGV 9* and proportion of Vγ9^+^ Vδ2^+^ T cells were identified as independent protective factors for complete remission in patients with AML.

**Conclusions:**

The restriction of *TRGV* usage might be related to the preference of usage of γδ T cells. Higher expression of *TRGV* subfamilies might be associated with better OS in AML. Higher *TRGV 9* expression and increased Vγ9^+^ Vδ2^+^ T cells subfamilies might indicate a better prognosis in patients with AML.

## Introduction

Acute myeloid leukemia (AML) is a malignant clonal disease originating from hematopoietic stem cells and characterized by genetic and clinical heterogeneity and high mortality (1). Despite considerable progress in treating hematological malignancies, clinical outcomes of patients older than 60 years are unfavorable, and the overall long-term survival in patients with AML remains poor (2). Recent studies have revealed that T cell immunodeficiency is a common characteristic of patients with AML, mainly due to peripheral T cells that restricted oligoclonal T cell repertoires, reduced thymic output function, and lower activation and response to antigens (3, 4).

T cells recognize specific ligands by specific T cell receptors (TCRs), which are heterodimers consisting of either αβ and γδ chains. Genes encode for the variable domains of *TRG* (γ chain) and *TRD* (δ chain), which are assembled by somatic recombination from variable (V), diversity (D, only for *TRD*), and joining (J) segments and compose three hypervariable or complementarity-determining regions (CDR1, CDR2, and CDR3) that occur during T cell differentiation (5, 6). The *TRG* gene contains several different functional variable (*TRGV*) segments belonging to four subgroups (*TRGV I–IV*), and the *TRD* gene contains at least eight functional *TRDV* segments that are subdivided into eight *TRDV* subfamilies (*TRDV* 1–8) (5–9). Previous studies showed that *TRGV IV* was a pseudogene, which was a simple combination between *TRGV IV* and *TRGC* segment lacking *TRGJ* segment and there was no any rearrangement in CDR3 by sequencing (10, 11). Hence, the analysis of *TRGV* repertoire was acquired in three *TRGV* subfamilies in the present study. Nowadays, according to their *TRD* (TCRδ) chain usage, human γδ T cells are mainly divided into 2 major subsets including Vδ1 and Vδ2 in peripheral blood (PB). Several functional *TRG* (TCRγ) gene segments are generally divided into Vγ2, γ3, γ4, γ5, γ8, γ9, γ10 (also termed *TRGV 2, TRGV 3, TRGV 4, TRGV 5, TRGV 8*, *TRGV 9* and *TRGV10*, respectively) (12, 13). The V-genes of *TRGV 2-5 and TRGV 8* have a relatively high sequence similarity, which are different from *TRGV 9* sequences. Different TCRγ chains and TCRδ chains can be combined to form different types of γδ T cells (14). Although Vδ1 T cells are predominantly associated with the Vδ1 comprising *TRGV 2, TRGV 3, TRGV 4, TRGV 5, TRGV 8*, which belonging to *TRGV I* subsets, the majority of Vδ2 T cells express an invariant TCR harboring *TRGV 9*, which belonging to *TRGV II* subsets (15). In addition, *TRGV 10* belongings to *TRGV III* subsets (12). In the PB of healthy individuals (HIs), there is a predominant expression in the γδ T cell population, which is the cell expressing Vγ9 together with Vδ2, termed Vγ9^+^ Vδ2^+^ T cells (15, 16). The roles of some T cell subgroups in cancer are controversial because they have been suggested to play both an anti-tumor role and a pro-tumor role. The heterogeneous T cells in AML have the combinatorial variety generated by different TCRs, which might explain why some special T cell subsets have a controversial role in cancer immunity. Although PD-1^+^Vβ5.2^+^ and PD-1^+^Vβ12^+^ CD8^+^ T cells were thought to be related to poor prognosis in AML (17), our previous study found that clonally expanded *TRDV* T cells might benefit the immune response directed against AML (18). However, the features of the *TRGV* repertoire in AML remain unknown, and the cellular immunity characteristics of AML have yet to be fully elucidated. To further understand the heterogeneity of γδ T cells, in this study, we first analyzed the distribution pattern and clonality of *TRGV* subfamilies and further investigated correlation between expression levels of *TRGV* subfamilies and proportion of Vγ9^+^ Vδ2^+^ T cells and their clinical relevance in patients with AML.

## Materials And Methods

### Samples

PB samples were collected from 75 patients with *de novo* AML (42 males and 33 females, median age 48 years, range 18–88 years) from January 2015 to December 2021. A total of 51 HIs (29 males and 22 females, median age 45 years, range 25–83 years) served as controls. Among the total samples, there were 56 patients with AML and 33 HIs were used to analyze the expression levels of *TRGV* subfamilies. Of the 56 patients identified, 50 patients with both available *TRGV* gene expression data and outcome information were eventually included in the survival analysis. In addition, the PB of extra 19 patients with AML and 18 HIs were analyzed by flow cytometry. Of the 19 patients, 18 patients with both available flow cytometry data and outcome information were also included in survival analysis. The clinical information was showed in **Table 1** and **Supplementary Table 1**. Informed consent was obtained from all participants. The protocol of all experiments was approved by the Ethics Committee of First Affiliated Hospital, Medical School of Jinan University.

**Table 1:** Clinical characteristics of AML patients.

Factor	AML
Number	75
Age (median; range)	48 (18-88)
Gender (Male/Female)	42/33
WBC (×10^9^/L), (median; range)	23.10 (1-325.42)
RBC (×10^12^/L), (median; range)	2.51 (1.28-5.67)
PLT (×10^9^/L), (median; range)	44.1 (4-632)
BM blast cells (%), (median; range)	63 (20-94)
FAB subtype (n=75)	
M0	6
M1	2
M2	16
M3	12
M4	9
M5	18
M6	/
M7	/
Undetermined	12
Gene mutation	
*FLT3* (+/-)	12/63
*NPM1* (+/-)	9/66
*PML/RARA* (+/-)	8/67
*MLL* (+/-)	6/69
*TP53* (+/-)	4/71
*AML1/ETO* (+/-)	5/70
Others (+/-)	9/66
Unknown (+/-)	29/46
Cytogenetic abnormality	
Normal (+/-)	6/69
Abnormal (+/-)	25/50
Unknown (+/-)	44/31
Treatment	
Chemotherapy (+/-)	66/9
HSCT (+/-)	9/66

AML, acute myeloid leukemia; WBC, white blood cell; RBC, red blood cell; PLT, platelet; BM blast cells, bone marrow blast cells; FAB, French-American-British; M0, minimally differentiated AML; M1, AML without maturation; M2, AML with maturation; M3, acute promyelocytic leukemia; M4, acute myelomonocytic leukemia; M5, acute monocytic leukemia; M6, pure erythroid leukemia; M7, Acute megakaryoblastic leukemia; HSCT, hematopoietic stem cell transplantation; /, unknown.

### Mononuclear Cell Isolation and γδ T Cell Sorting

The Ficoll–Hypaque gradient centrifugation method was used to isolate mononuclear cells from fresh PB. The γδ T cells were sorted by γδ monoclonal antibodies and MACS magnetic cell sorting technique (Miltenyi Biotec, Germany) (19). All samples were freshly obtained and subjected to immediate preparation.

### RNA Isolation and cDNA Synthesis

According to the manufacturer’s recommendations, total RNA of γδ T cells was extracted by Trizol (Invitrogen, USA). Superscript II Kit (Gibco, USA) was used to synthesize the first single-strand complementary DNA (cDNA). Subsequently, the quality of cDNA was confirmed by RT-PCR for *β*2 microglubin (*β*2M) gene amplification (the primers of *β*2M gene for RT-PCR were list in **Table 2**) (20).

**Table 2:** Sequences of primers used in RT-PCR and qPCR.

Primer	Sequence
*TRGV I*	5’-TACCTACACCAGGAGGGGAAG-3’
*TRGV 9*	5’-GGCACTGTCAGAAAGGAATC-3’
*TRGV III*	5’-TCGACGCAGCATGGGTAAGAC-3’
Cγ	5’- GTTGCTCTTCTTTTCTTGCC-3’
Cγ-FAM	5’-FAM-CATCTGCATCAAGTTGTTTATC-3’
*β*2M-for	5’-TACACTGAATTCACCCCCAC-3’
*β*2M-back	5’-CATCCAATCCAAATGCGGCA-3’

### RT-PCR for *TRGV* Subfamily Amplification and Genescan Analysis for *TRGV* Subfamily Clonality Analysis

Three sense *TRGV* primers and a single *TRGC* reverse primer were used in unlabeled PCR for the amplification of the *TRGV* subfamilies. Runoff PCR was performed with fluorescent primers labeled at the 5’ end with the FAM fluorophore (Cγ-FAM) (TIB MOLBIOL GmbH, Germany). A DNA thermal cycler (BioMetra, Germany) was used to perform this reaction process. The primers are listed in **Table 2**. PCR was performed as described in our previous report (19–21). Aliquots of cDNA (1 μL) were amplified in 20 μL reactions with one of the three Vγ primers and a Cγ primer. The final reaction mixture contained 0.5 μM sense primer and antisense primer, 0.1 mM dNTPs, 1.5 mM MgCl_2_, 1× PCR buffer, and 1.25 U Taq polymerase (Promega, USA). After 3 min of denaturation at 94°C, 40 PCR cycles were carried out (94°C for 1 min, 60°C for 1 min, and 72°C for 1 min and a final elongation for 6 min at 72°C). All PCR products were stored at 4°C and ready for Genescan analysis (22).

Aliquots of the unlabeled PCR products (2 μL) were subjected to a cycle of runoff reaction with a fluorophore-labeled Cγ-FAM primer. The labeled runoff PCR products (2 μL) were heat-denatured at 94°C for 4 min with 9.5 μL of formamide (Hi-Di Formamide, ABI, USA) and 0.5 μL of size standards (GENESCAN™-500-LIZ™, Perkin Elmer, USA). The samples were then loaded on 3100 POP-4™ gel (Performance Optimized Polymer-4, ABI, USA) and resolved by electrophoresis in an ABI 3100 DNA sequencer for size and fluorescence intensity determination using Genescan software (23).

### Real-Time Quantitative PCR (qPCR) for *TRGV* Gene

The gene expression levels of the *TRGV* subfamilies in cDNA of γδ T cells were determined by qPCR with SYBR Green I technique, and the *β*_2_-microglobulin (*β*_2_M) gene was used as an endogenous reference. The primers are listed in **Table 2**. qPCR was performed as described by Stams WAG et al. and our previous study (10, 24–26). In brief, qPCR was performed in a total volume of 20 μL with approximately 1 μL cDNA, 0.5 μM of each primer (one of the three *TRGV* sense primer and the antisense primer Cγ for *TRGV* amplification, *β*_2_M-for and *β*_2_M-back primers for *β*_2_M gene amplification), 2x RealMastrMix 10 μL (Tiangen, China). After 2 min of denaturation at 95°C, 40 PCR cycles were carried out (95°C for 15 s, 58°C for 20 s, and 72°C for 30 s). At the end of each run, melting curve analysis was performed starting at 65°C up to 95°C with an increase of 1°C per 2 s to verify primer specificities. Specific amplification of PCR products was analyzed by melting curve analysis. qPCR was repeated in at least three separate experiments. The following equation was used to calculate the relative expression level to the *β*_2_M gene for each target PCR. Relative mRNA expression = 2^-ΔCt^ × 100% [ΔC_t_ = C_t(_*_TRGV_*_subfamilies)_ – C_t(_*_β_*_2M)_] (15).

### Flow Cytometry

The following monoclonal antibodies APC/Cy7 anti-human CD3 (clone SK7), PE/Cy7 anti-human TCR γ/δ (clone B1), PerCP anti-human TCR Vδ2 (clone B6), and APC anti-human TCR Vγ9 (clone B3; Biolegend, USA) were used for cell surface staining following the manufacturer’s instructions (27). The stained cells were examined with BD FACS VERSE flow cytometer (BD, USA), and data were analyzed by Flowjo software (Flowjo LLC, USA).

### Statistical Analysis

In this study, data were presented as median. Fisher’s exact test was used to compare expression frequencies of three *TRGV* subfamilies between AML patients and HIs. Kruskal–Wallis test was used for comparison of different gene expression levels from different *TRGV* subfamilies in AML and HIs. Differences in mRNA expression level of *TRGV* between two groups were analyzed using the Mann–Whitney U test. Pearson correlation analysis was used to analyze the correlation of mRNA expression levels of *TRGV* subfamilies between two groups. Binary logistic regression analysis was performed to determine associations between expression levels of three *TRGV* subfamilies and clinical outcome of the AML patients. Through Kaplan-Meier method and cox regression analysis the effect of *TRGV* expression and the proportion of Vγ9^+^ Vδ2^+^ T cells on prognosis of AML were analyzed. All analyses included the following variables: including gender, age, white blood cell (WBC), red blood cell (RBC), platelet (PLT), bone marrow (BM) blast cells, French-American-British (FAB) subtype, gene mutation and treatment in patients. Only values with *P* < 0.05 was regarded as statistically significant. All results were analyzed by SPSS 25.0 and GraphPad Prism 8.4.

## Results

### Expression Pattern and Clonality of the *TRGV* Repertoire in Patients With *De Novo* AML

**Figure 1:**
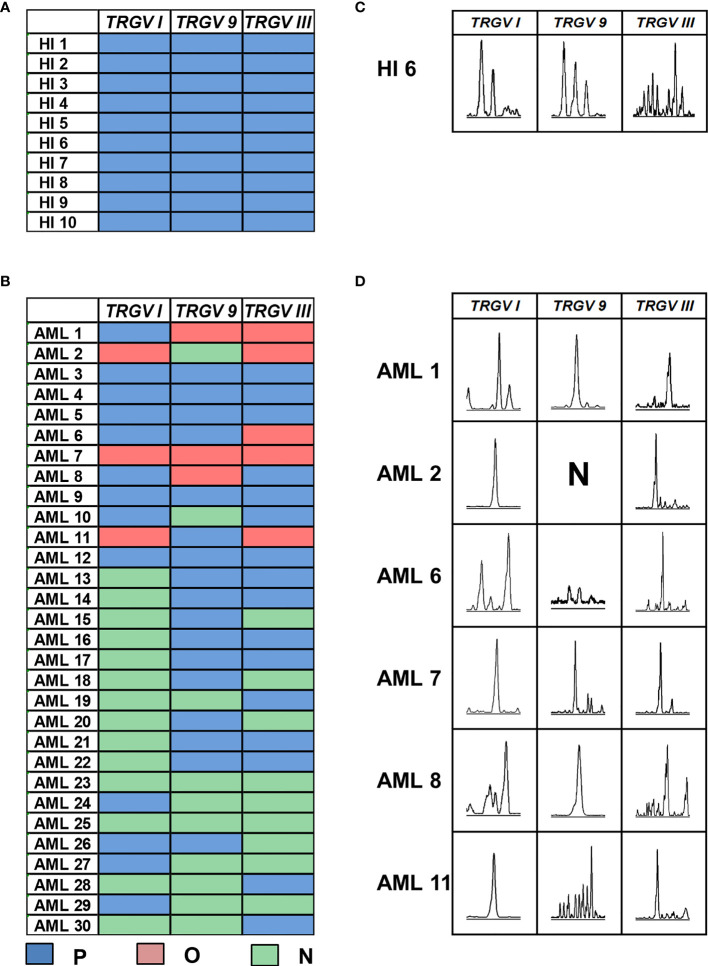
Distribution and clonality of *TRGV* subfamilies in γδ T cells from 10 healthy individuals and 30 patients with AML. The feature of distribution and clonality of *TRGV* subfamilies in healthy individuals and patients with AML **(A, B)**. Expression pattern of *TRGV* subfamilies in one case of healthy individual and six AML patients **(C, D)**. P, polyclonality; O, oligoclonality; N, negative.

**Figure 2:**
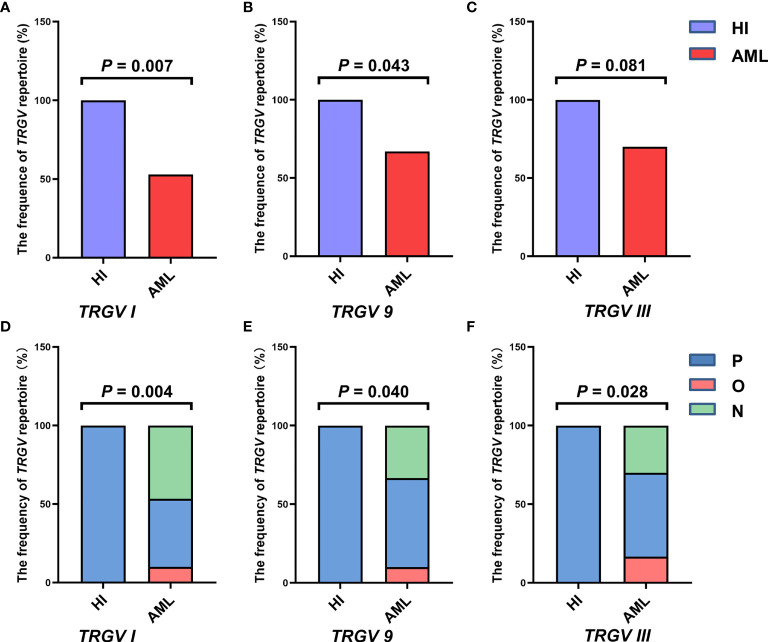
Frequencies of the *TRGV* subfamilies in γδ T cells from 10 healthy individuals and 30 patients with AML. The expression frequencies of three *TRGV* subfamilies in healthy individuals and patients with AML (using the Fisher’s exact test) **(A–C)**. The clonal expansion frequency of the three *TRGV* subfamilies in healthy individuals and patients with AML (using the Fisher’s exact test) **(D–F)**. P, polyclonality; O, oligoclonality; N, negative.

In this study, the CDR3 region of three *TRGV* subfamily genes was analyzed by Genescan analysis in γδ T cells from 30 patients with *de novo* AML and 10 HIs to assess the spectral pattern visually. Diversity and clonality of TCR repertoire demonstrated the ability of specific amplifications to respond to antigen stimulation. Based on the CDR3 TCR rearrangement lengths, the clonality of γδ T cells was characterized as multipeaks and oligopeaks responding to polyclonality and oligoclonality. Polyclonality of the *TRGV* subfamily genes displayed a Gaussian distribution consisting of three or more peaks, and oligoclonality was a skewed spectral profile showing a single dominant peak. In this study, all patients with AML had a significantly skewed TCR repertoire with 16–21 of the three *TRGV* subfamilies (*TRGV I*, *9*, and *III*) detected in each patient. Among AML samples, the most frequently expressed subfamily members were *TRGV III* (70%, 21/30) and *TRGV 9* (66.67%, 20/30). *TRGV I* from patients with AML was detected only in 16 cases (53.33%, 16/30; **Figures 1A–D**). All of the three *TRGV* subfamilies could be detected in γδ T cells from HIs. The expression frequencies of the *TRGV I* and *TRGV 9* subfamilies in patients with AML were lower than those in HIs (*TRGV I*: *P* = 0.007, *TRGV 9*: *P* = 0.043), whereas the *TRGV III* subfamily in AML was similar to that in HIs (*P* = 0.081; **Figures 2A–C**).

The deviation from the Gaussian profile could indicate a clonally expanded pattern. The PCR products produced only one peak, which represented that CDR3 lengths were identical, named oligoclonal pattern. We further analyzed the different clonotypic expansion patterns in HIs and patients with AML. Oligoclonal expansion was detected in the *TRGV* subfamily from six out of 30 cases in patients with AML (**Figure 1D**). The expression frequencies of clonally expanded *TRGV* subfamilies in the patients with AML were as follows: *TRGV III* (17%, 5/30), *TRGV I* (10%, 3/30), and *TRGV 9* (10%, 3/30). However, there were no clonally expanded *TRGV* subfamilies that could be identified in HIs. Based on the clonally expanded pattern, we divided the clonal expansion frequency of the three *TRGV* subfamilies into three groups: polyclonality, oligoclonality and negative groups. The results showed a significant difference between patients with AML and HIs, and the clonal expansion frequencies of the *TRGV* subfamilies were statistically higher than those of HIs (*TRGV I*: *P* = 0.004; *TRGV 9*: *P* = 0.040; and *TRGV III*: *P* = 0.028; **Figures 2D–F**).

### Gene Expression Level of the *TRGV* Subfamily

**Figure 3:**
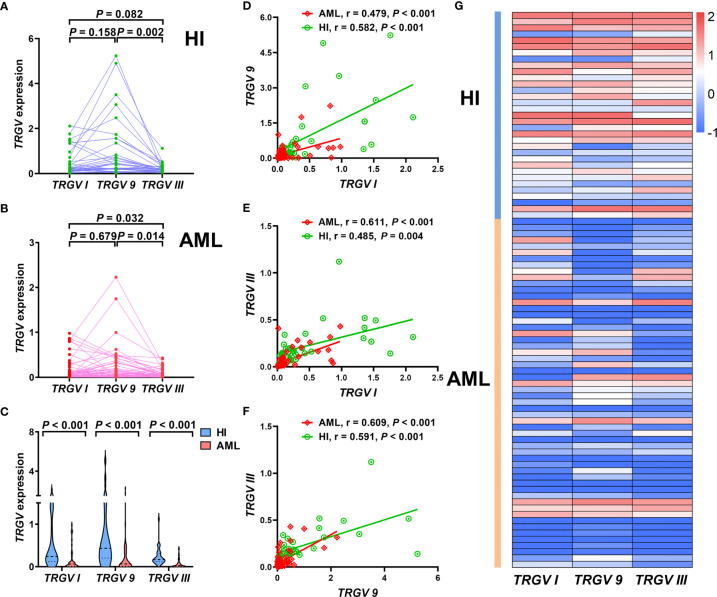
Pattern of expression levels of three *TRGV* subfamilies in γδ T cells from 33 cases with healthy individuals and 56 cases with AML (using the Mann Whitney test) **(A–C)**. Correlations among three *TRGV* subfamilies in 33 healthy individuals and 56 patients with AML (using the Pearson correlation analysis) **(D–F)**. Heatmap representing the expression levels of three *TRGV* subfamilies in 33 healthy individuals and 56 patients with AML **(G)**.

Subsequently, we focused on detecting expression levels of *TRGV* subfamilies by qPCR, so we expanded the samples’ quantity, and further collected extra 26 AML samples on the basis of the original 30 samples. Therefore, three *TRGV* genes expression levels in a total of 56 patients with AML and 33 HIs as control were detected in our study. Results showed significant differences of expression levels in the *TRGV* subfamilies of HIs (*χ*^2^ = 9.998, *P* = 0.007) between *TRGV I* and *TRGV 9* (*P* = 0.158), *TRGV 9* and *TRGV III* (*P* = 0.002), and *TRGV I* and *TRGV III* (*P* = 0.082; **Figure 3A**). There were also significant differences in the *TRGV* subfamilies of AML (*χ*^2^ = 7.208, *P* = 0.027) between *TRGV I* and *TRGV 9* (*P* = 0.679), *TRGV 9* and *TRGV III* (*P* = 0.014), and *TRGV I* and *TRGV III* (*P* = 0.032; **Figure 3B**). We further compared the gene expression levels of the *TRGV* subfamilies in patients with AML and HIs. The gene expression levels of the three *TRGV* subfamilies in AML were lower than those in HIs (*P* < 0.001, *P* < 0.001, and *P* < 0.001; **Figures 3C, G**).

We also obtained more insight to investigate the correlation of the gene expression levels of the three *TRGV* subfamilies in HIs and patients with AML. In HIs, a significant positive correlation was found in the expression levels of *TRGV I* and *TRGV 9* (*r* = 0.582, *P* < 0.001), *TRGV I* and *TRGV III* (*r* = 0.485, *P* = 0.004), and *TRGV 9* and *TRGV III* (*r* = 0.591, *P* < 0.001; **Figures 3D–F**). A positive correlation in the expression levels of *TRGV I* and *TRGV 9* (*r* = 0.479, *P* < 0.001), *TRGV I* and *TRGV III* (*r* = 0.611, *P* < 0.001), and *TRGV 9* and *TRGV III* (*r* = 0.609, *P* < 0.001) was also observed in patients with AML (**Figures 3D–F**).

### Proportions of Total γδ T Cells and Vγ9^+^ Vδ2^+^ Subsets in patients With AML

**Figure 4:**
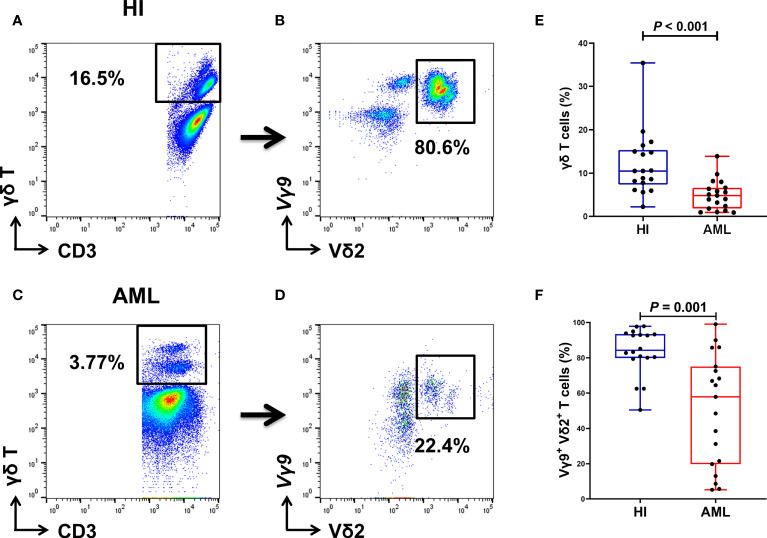
Gating strategy for identifying the percentage of γδ T cells from PB in 18 HIs and 19 patients with AML. Flow cytometry detection of the percentage of CD3^+^ γδ T cells and Vγ9^+^ Vδ2^+^ T cells in HIs **(A, B)** and patients with AML **(C, D)**. The percentage of γδ T cells in HIs and patients with AML (using the Mann Whitney test) **(E)**. Comparison of the percentages of Vγ9^+^ Vδ2^+^ T cells in HIs and patients with AML (using the Mann Whitney test) **(F)**.

Based on previous finding, we were more interested in proportions of total γδ T cells and Vγ9^+^ Vδ2^+^ T cell subsets from PB, so another 19 AML samples and 18 HIs were further collected and analyzed for FACS (**Figures 4A–D**). Compared with HIs, significantly lower proportions of total γδ T cells (median: 4.83% vs. 10.5%) and Vγ9^+^ Vδ2^+^ T cells (median: 57.9% vs. 84.25%) were found in patients with AML (*P* < 0.001 and *P* = 0.001, respectively; **Figures 4E, F**).

### *TRGV* Repertoire and Its Clinical Relevance in AML

Despite the increased insight into the phenotype of γδ T cells, whether it correlates with clinical outcome remains poorly understood. To further understand the role of the *TRGV* subfamily and the prognosis of patients with AML, we analyzed correlation between the expression levels of *TRGV* subfamily genes and the frequency of Vγ9^+^ Vδ2^+^ T cells with the clinical prognosis of AML. We first focused on whether expression levels of *TRGV* subfamily genes affected AML clinical prognosis and assessed the clinical prognosis of the 56 AML patients. Due to 6 patients who refused therapy and voluntarily left the hospital, we finally analyzed the prognosis and outcome of 50 AML patients. Univariate and multivariate logistic regression analysis were used to analyze the expressive levels of three *TRGV* subfamilies and other impact factors, including gender, age, WBC, RBC, PLT, BM blast cells, AML subtype, gene mutation and treatment in patients with AML. The patients who followed up after first-cycle chemotherapy were divided into complete remission (CR) and non-CR groups based on BM smears and flow cytometry analysis. Univariate logistic regression analysis demonstrated high WBC counts was an independent risk factor for CR (*P* = 0.023, odds ratio (OR) = 1.013, 95% confidence interval (CI): 1.002-1.024), whereas high expression levels of *TRGV I*, *TRGV 9*, and *TRGV III* were the significant independent protective factors for CR (*TRGV I*: *P* = 0.012, OR = 0.211, 95% CI: 0.062-0.711; *TRGV 9*: *P* = 0.012, OR = 0.211, 95% CI: 0.062-0.711; *TRGV III*: *P* = 0.003, OR = 0.141, 95% CI: 0.039-0.504),. However, there was no significant difference in gender, age, RBC, PLT, BM blast cells, AML subtype, gene mutation and treatment (*P* > 0.05). Interestingly, multivariate logistic regression analysis showed that *TRGV 9* expression was an independent protective factor for CR (*TRGV 9*: *P* = 0.035, OR = 0.079, 95% CI: 0.007-0.831). Besides, we further used univariate and multivariate cox regression analysis to further analyze the relationship between those factors and overall survival (OS) in AML patients. Univariate cox regression analysis showed that high counts of WBC (*P* = 0.001, hazard ratio (HR) = 1.010, 95% CI: 1.004-1.015) and the AML subtype (non-M3) (*P* = 0.047, HR = 7.845, 95% CI: 1.028-59.865) was associated with unfavorable OS in AML patients. Importantly, high expression levels of *TRGV I*, *TRGV 9*, and *TRGV III* were associated with favorable OS in AML patients (*TRGV I*: *P* = 0.018, HR = 0.258, 95% CI: 0.084-0.794; *TRGV 9*: *P* = 0.004, HR = 0.111, 95% CI: 0.025-0.488; *TRGV III*: *P* = 0.028, HR = 0.283, 95% CI: 0.092-0.871). Multivariate cox regression analysis also showed that high *TRGV 9* expression could mark an improved OS in patients with AML (*TRGV 9*: *P* = 0.048, HR = 0.084, 95% CI: 0.007-0.979; **Table 3**).

**Table 3:** Univariate and multivariate logistic and cox regression analysis in AML patients.

Variables	Univariate logistic regression		Multivariate logistic regression		Univariate cox regression		Multivariate cox regression	
	OR	*P*-value	OR	*P*-value	HR	*P*-value	HR	*P*-value
	(95% CI)		(95% CI)		(95% CI)		(95% CI)	
Sex (reference male)								
Female	0.923 (0.297, 2.865)	0.890	0.212 (0.026, 1.697)	0.144	1.602 (0.617, 4.158)	0.333	0.957 (0.314, 2.919)	0.938
Age (year)	1.034 (0.999, 1.070)	0.059	1.064 (1.000, 1.134)	0.052	1.016 (0.985, 1.049)	0.309	1.002 (0.964, 1.042)	0.915
WBC, 10^9^/L	1.013 (1.002, 1.024)	0.023	1.010 (0.991, 1.029)	0.325	1.010 (1.004, 1.015)	0.001	1.009 (1.000, 1.018)	0.059
RBC, 10^12^/L	1.139 (0.599, 2.168)	0.691	4.044 (0.797, 20.528)	0.092	0.896 (0.512, 1.568)	0.700	3.049 (1.093, 8.503)	0.033
PLT, 10^9^/L	1.000 (0.994, 1.006)	0.975	1.001 (0.992, 1.010)	0.824	1.000 (0.995, 1.005)	0.984	1.004 (0.996, 1.012)	0.341
BM blast cell, %	1.002 (0.972, 1.033)	0.905	1.024 (0.974, 1.075)	0.354	1.007 (0.981, 1.034)	0.592	1.008 (0.973, 1.045)	0.653
FAB subtype (reference non-M3)								
M3-AML	4.275 (0.816, 22.390)	0.086	1.071 (0.045, 25.315)	0.966	7.845 (1.028, 59.865)	0.047	4.584 (0.197, 106.861)	0.343
Gene mutation (reference non-*FLT3* mutation)								
*FLT3* mutation	0.404 (0.073, 2.235)	0.299	0.126 (0.009, 1.711)	0.120	1.475 (0.48, 4.532)	0.497	1.937 (0.373, 10.060)	0.431
Treatment (reference chemotherapy)								
HSCT	0.800 (0.169, 3.793)	0.779	0.904 (0.110, 7.427)	0.925	0.438 (0.099, 1.932)	0.276	0.294 (0.032, 2.656)	0.275
*TRGV I*	0.211 (0.062, 0.711)	0.012	1.243 (0.086, 17.973)	0.873	0.258 (0.084, 0.794)	0.018	0.552 (0.026, 11.652)	0.703
*TRGV 9*	0.211 (0.062, 0.711)	0.012	0.079 (0.007, 0.831)	0.035*	0.111 (0.025, 0.488)	0.004	0.084 (0.007, 0.979)	0.048*
*TRGV III*	0.141 (0.039, 0.504)	0.003	0.069 (0.004, 1.161)	0.063	0.283 (0.092, 0.871)	0.028	1.221 (0.067, 22.238)	0.893

AML, acute myeloid leukemia; CR, complete remission; OS, overall survival; OR, odds ratio; 95% CI, 95% confidence interval; HR, hazard ratio; WBC, white blood cell; RBC, red blood cell; PLT, platelet; M3, acute promyelocytic leukemia; HSCT, hematopoietic stem cell transplantation. *P < 0.05.

Furthermore, we also used the same way to access the relationship between the proportion of γδ T cells, Vγ9^+^ Vδ2^+^ T cells and the prognosis of 19 AML patients. Univariate logistic regression analysis showed that the high proportion of Vγ9^+^ Vδ2^+^ T cells was an independent protected factor for CR (*P* = 0.044, OR = 0.963, 95% CI: 0.927-0.999), and age was an independent risk factor for AML-CR (*P* = 0.035, OR = 1.128, 95% CI: 1.009-1.261), but there was no significant difference in γδ T cells and other factors (gender, age, WBC, RBC, PLT, BM blast cells, AML subtype, gene mutation and treatment) (*P* > 0.05) (data were not showed). Due to insufficient numbers of AML samples, there was no significant difference in multivariate logistic regression analysis. There was one patient was voluntarily left the hospital because of impact of COVID-19 in total 19 AML patients, so we collected outcome of 18 AML patients. Univariate cox regression analysis showed that patients with high proportion of γδ T cells had low risk of death than those with low proportion (*P* = 0.008, hazard ratio (HR) = 0.109, 95% CI: 0.021-0.564), while multivariate cox regression analysis showed no significant difference (*P* > 0.05) (data were not showed).

### The Relationship Between *TRGV* Expression and Prognosis in AML Patients

**Figure 5:**
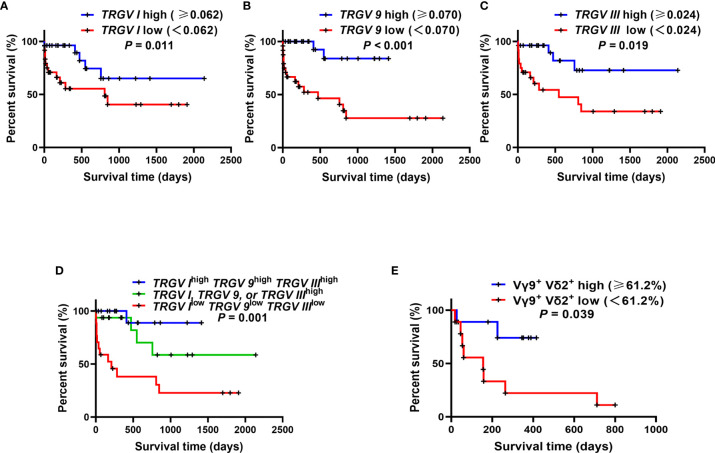
Overall survival (OS) analysis of the gene expression levels of three *TRGV* subfamilies in 50 AML patients and the percentages of Vγ9^+^ Vδ2^+^ T cells in 18 AML patients. Kaplan–Meier curves showed the OS for the high (blue line) and low (red line) *TRGV* expression groups **(A–C)**. Kaplan–Meier curves showed the OS for the co-high expression (blue line), single high expression (green line), and co-low expression (red line) of *TRGV I*, *TRGV 9* and *TRGV III* **(D)**. Kaplan–Meier curves showed the OS for the high (blue line) and low (red line) percentages of Vγ9^+^ Vδ2^+^ T cells **(E)**.

The survival analysis demonstrated that the high expression levels of *TRGV I, TRGV 9* and *TRGV III* were significant related to better OS (*P* = 0.011; *P* < 0.001; *P* = 0.019) (**Figures 5A–C**). To better understand the combination of three *TRGV* subfamilies in predicting the OS of AML patients, we divided patients into the following 3 groups: *TRGV I*^high^ *TRGV 9*^high^ *TRGV III*^high^, *TRGV I, TRGV 9*, or *TRGV III*^high^ and *TRGV I*^low^ *TRGV 9*^low^ *TRGV III*^low^. Interestingly, the results suggested that the group of *TRGV I*^high^ *TRGV 9*^high^ *TRGV III*^high^ had longer survival time (*P* = 0.001) (**Figure 5D**). Next, we further access the proportion of Vγ9^+^ Vδ2^+^ T cells from PB with the clinical outcome of AML patients. The OS in high Vγ9^+^ Vδ2^+^ T cells were longer than those in low Vγ9^+^ Vδ2^+^ T cells group (*P* = 0.039) (**Figure 5E**).

## Discussion

The attractive features of γδ T cells include non-MHC-restricted antigen recognition and abundant cytokine secretion capacity, which have raised expectations for their application in cancer adoptive immunotherapy (28–30). The combinatorial variety generated by different TCRs might be the reason why γδ T cells can exert diverse actions in distinct pathological types of diseases (31). T cell immunodeficiency is a common feature in different hematological malignancies, including AML, immune thrombocytopenic purpura (ITP), B cell non-Hodgkin lymphoma, and graft-versus-host disease (GVHD) (21, 32–34). Analysis of alterations in the TCR repertoire is a practical approach that can help understand the involved immunological abnormalities and provide guidance for clinics in translational research (19). Analysis of the *TRGV* and *TRDV* repertoire provides a global picture of the distribution and clonal expansion of TCR γδ subfamilies in ITP, multiple myeloma (MM), and GVHD (21, 32, 35, 36). Our previous study also showed the clonally expanded *TRDV* T cells in AML (18). However, the features of the *TRGV* repertoire in AML remain unknown.

In this study, we investigated the expression pattern of TCR Vγ (*TRGV*) subfamilies and characterized the correlation between the expression of *TRGV* and clinical outcome in patients with AML. To further compare the difference in TCR repertoire diversity, three *TRGV* gene spectral profiles were examined by Genescan analysis. In HIs, polyclonal expanded T cells, which showed a small proportion of multiple peaks, were detected in the majority of the *TRGV* subfamily. By contrast, a clonotypic expansion pattern, which included a high peak together with one or a few lower peaks named oligoclonality, was a common pattern for each sample. Skewed expression of the *TRGV* repertoire was an obvious characteristic of patients with AML compared with HIs who expressed nearly all of the *TRGV* subfamilies, which indicated that patients with AML might have low diverse immune responses due to γδ T cell immunodeficiency. The T cell spectra are commonly characterized by a Gaussian distribution containing 6–8 peaks, which are named polyclonality in HIs, representing a repertoire that guarantees sufficiently diverse T cell clones (37). The clonally expanded T cell repertoire was also detected in all samples in this study. Multiple oligoclonal expanded *TRGV* subfamilies were demonstrated in patients with AML who were different from HIs. Thus, the oligoclonal *TRGV* repertoire might be associated with leukemia-associated antigen.

We also found that the gene expression levels of the *TRGV* repertoire in γδ T cells between AML and HIs were different, and lower expression levels were found in *TRGV* genes in AML than in HIs. The change and pattern of *TRGV* subfamilies demonstrated that restrictive *TRGV* usage might be related to the preference of usage of γδ T cells. The biological significance of the difference observed remains unknown, so we attempted to characterize the association between the expression level of the *TRGV* repertoire and clinical patient characteristics. Our previous study showed that TIGIT^+^ Foxp3^+^ γδ T cells and TIGIT^+^ CD226^-^ γδ T cells were related to the clinical outcome of patients with AML (38, 39). In the present study, we further analyzed the relationship between the expression of the *TRGV* repertoire and the OS of patients with AML. Our results showed that a higher expression level of *TRGV* subfamilies was associated with better OS in patients with AML, and patients with highly *TRGV I*, *TRGV 9*, and *TRGV III* genes co-expressed had better OS than their counterparts. Moreover, we found that *TRGV 9* was an independent protective factor in AML-CR, thereby indicating that patients with high *TRGV 9* expression may have the better prognosis than those with low expression. In addition, our data showed that increased Vγ9^+^ Vδ2^+^ T cells subfamilies in patients with AML might correlate with better therapeutic effects. Related research showed that γδ T cells played an essential role in cancer (40). Such cells have a long-term disease-free survival advantage to patients with AML and increased γδ T cells following hematopoietic stem cell transplantation (HSCT) (41, 42). The known pleiotropic effects of γδ T cells suggest multiple mechanisms by which γδ T cells might promote survival after HSCT, which were consistent with our findings in patients with AML. Understanding the characteristics of *TRGV* subsets in patients with AML may be helpful for clinical application and promote the treatment of patients. However, these γδ T cell subfamilies exerted certain anti-leukemia effects, so the anti-leukemia potency of γδ T cells could be exhausted due to prolonged antigenic stimulation. In the long run, we should choose a specific anti-tumor γδ T cell subgroup in γδ T cell immunotherapy and try to use a combination of γδ T cell adoptive immunotherapy and immune checkpoint inhibitors.

## Conclusion

Taken together, in addition to the previously reported clonally expanded *TRDV* T cells in AML (18), our data further provide a detailed profile and feature of the *TRGV* repertoire in patients with AML. Importantly, the patients with AML who had high expression level of the *TRGV* gene or higher proportion of Vγ9^+^ Vδ2^+^ T cells were associated with favorable OS, which may be related to resorting anti-AML γδ T function. Further studies are required to confirm and dissect the detailed mechanisms. These findings could partially explain to promote our understanding of the cellular immune features of γδ T cells, which brings hope for immunotherapy to treat AML patients.

## Data Availability Statement

The original contributions presented in the study are included in the article/**Supplementary Material**. Further inquiries can be directed to the corresponding authors.

## Ethics Statement

The protocol of all experiments was approved by the Ethics Committee of the First Affiliated Hospital of Jinan University. The patients/participants provided their written informed consent to participate in this study. Written informed consent was obtained from the individual(s) for the publication of any potentially identifiable images or data included in this article.

## Author Contributions

XW and ZJ were involved in experimental design and the concept development. XK and XL conducted the experiments. WW and XJ contributed to data analysis and figure preparation. JC and JL provided all samples and clinical data. ZJ, XK, and XW drafted the manuscript. All authors read and approved the final manuscript.

## Funding

This study was supported by grants from the National Natural Science Foundation of China (Nos. 81800143, 81770150, 81200388, and 82170220), Natural Science Foundation of Guangdong Province (No. 2018A0303130220 and 2020A1515010817), the Science and Technology Planning Project of Guangzhou City of China (No. 201804010425), Medical Scientific Research Foundation of Guangdong Province (No. A2018565 and A2017198), Special Funds for the Cultivation of Guangdong College Students’ Scientific and Technological Innovation (No. 202010559078), and Guangdong College Students’ Scientific and Technological Innovation (Nos. CX21283, CX21285, and CX20137).

## Conflict of Interest

The authors declare that the research was conducted in the absence of any commercial or financial relationships that could be construed as a potential conflict of interest.

## Publisher’s Note

All claims expressed in this article are solely those of the authors and do not necessarily represent those of their affiliated organizations, or those of the publisher, the editors and the reviewers. Any product that may be evaluated in this article, or claim that may be made by its manufacturer, is not guaranteed or endorsed by the publisher.

## Abbreviations

AML, acute myeloid leukemia; TCRs, T cell receptors; CDR, complementarity-determining region; PB, peripheral blood; HIs, healthy individuals; *β*_2_M, *β*_2_ microglubin; qPCR, real-time quantitative PCR; WBC, white blood cell; RBC, red blood cell; PLT, platelet; BM, bone marrow; FAB, French-American-British; CR, complete remission; OR, odds ratio; CI, confidence interval; OS, overall survival; HR, hazard ratio; ITP, immune thrombocytopenic purpura; GVHD, graft-versus-host disease; MM, multiple myeloma; HSCT, hematopoietic stem cell transplantation.
