# Supplementary material for: High TRGV 9 Subfamily Expression Marks an Improved Overall Survival in Patients With Acute Myeloid Leukemia
Source: Front Immunol. 2022 Feb 10;13:823352. doi: 10.3389/fimmu.2022.823352 (PMC8866455; doi:10.3389/fimmu.2022.823352)
Supplement: Supplementary file 1 [file Table_1.doc]

**Supplementary table I Clinical information for the AML patients**

| NO. | F/M | Age | Subtype | WBC (10^9^/L) | RBC (10^12^/L) | PLT (10^9^/L) | BM blast cells(%) | Genetype | Therapy | Disease status | OS (days) |
| --- | --- | --- | --- | --- | --- | --- | --- | --- | --- | --- | --- |
| AML1 | F | 48 | M2 | 266.08 | 2.27 | 86 | 50 | ND | MA | non-CR | 163 |
| AML2 | M | 54 | M0 | 2.59 | 3.56 | 222 | 68.5 | ND | HSCT | non-CR | 756 |
| AML3 | F | 23 | M0 | 7.03 | 4.64 | 89 | 52 | ND | MA | CR | / |
| AML4 | F | 23 | M4 | 54.04 | 2.12 | 39 | 88 | FLT3 | MA | CR | 847 |
| AML5 | M | 30 | M2 | 7.25 | 2.33 | 24 | 78 | AML1-ETO | IA | CR | 334 |
| AML6 | F | 68 | M0 | 30.18 | 1.86 | 49 | 29 | ND | MA | non-CR | / |
| AML7 | M | 35 | M3 | 23.1 | 3.03 | 20 | 88 | PML-RARA | ATRA-MIT | CR | 822 |
| AML8 | M | 18 | M3 | 13.56 | 2.31 | 49 | 39 | PML-RARA | ATRA-ATO, IDA | CR | 862 |
| AML9 | F | 18 | M3 | 31.84 | 2.77 | 14 | 75.5 | PML-RARA | ATRA | non-CR | 1 |
| AML10 | F | 49 | M5 | 2.86 | 2.77 | 58 | 39 | ND | DA | CR | 179 |
| AML11 | M | 18 | M5 | 5.6 | 4.24 | 104 | 75.5 | ND | MA | CR | 71 |
| AML12 | F | 34 | M5 | 5.51 | 4.28 | 61 | 75 | MLL | HSCT | non-CR | 471 |
| AML13 | M | 67 | M2 | 54.89 | 3.32 | 12 | 87 | NPM1 | MA | non-CR | 283 |
| AML14 | F | 54 | M3 | 1.12 | 1.47 | 20 | 47 | ND | ATRA-ATO | CR | 788 |
| AML15 | M | 36 | M4 | 64 | 3.98 | 36.5 | 53 | ND | MA | CR | / |
| AML16 | F | 53 | M3 | 2.2 | 2.5 | 51 | 62 | FLT3 | HSCT | CR | 1910 |
| AML17 | M | 23 | M4 | 59.54 | 2.45 | 24.4 | 91 | ND | HSCT | CR | 2141 |
| AML18 | M | 29 | M3 | 80.4 | 3.58 | 38 | 82 | ND | HSCT | non-CR | 1291 |
| AML19 | M | 68 | M0 | 28.32 | 2.54 | 40 | 40 | ND | MA | non-CR | 34 |
| AML20 | M | 35 | M0 | 21.11 | 1.28 | 12 | 56.5 | ND | MA | non-CR | / |
| AML21 | F | 34 | M2 | 28.73 | 3.89 | 27 | 43 | ND | HSCT | CR | 1006 |
| AML22 | F | 33 | M0 | 50.64 | 3.78 | 118 | 60 | EVI1 | MA | non-CR | 549 |
| AML23 | M | 77 | M2 | 28.24 | 2.31 | 29 | 65.5 | ND | MA | non-CR | / |
| AML24 | F | 42 | M3 | 14.53 | 2.55 | 21.2 | 60 | PML-RARA | ATRA-ATO | CR | 566 |
| AML25 | M | 41 | M5 | 14.48 | 2.2 | 9.4 | 63 | MLL/AF6, EVI1 | MA | non-CR | 35 |
| AML26 | M | 30 | M3 | 2.33 | 3.54 | 31 | 84 | PML-RARA | ATRA-ATO-MIT | CR | 1227 |
| AML27 | F | 30 | M3 | 5.16 | 2.32 | 16 | 81 | PML-RARA | ATRA-ATO | CR | 1413 |
| AML28 | M | 50 | M1 | 135.81 | 1.57 | 9 | 85 | FLT3-ITD, NPM1 | HSCT | CR | 276 |
| AML29 | M | 44 | M5 | 94.19 | 3.47 | 89 | 62 | EVI1 | HSCT | CR | 349 |
| AML30 | F | 48 | M3 | 6.51 | 2.38 | 12 | 63 | ND | ATRA | non-CR | / |
| AML31 | F | 70 | M2 | 139.21 | 1.74 | 16 | 64 | ND | MA | non-CR | 67 |
| AML32 | M | 60 | M5 | 4.61 | 3.4 | 125 | 62.5 | MYC | IA | CR | 1414 |
| AML33 | M | 18 | M2 | 4.68 | 2.5 | 345 | 80 | C-Kit/D816V | MA | CR | 409 |
| AML34 | M | 53 | M2 | 5.17 | 3.15 | 490 | 51 | ND | MA | non-CR | 172 |
| AML35 | F | 50 | M5 | 3.24 | 2.55 | 252 | 85 | DNMT3A | IA | CR | 184 |
| AML36 | M | 47 | M5 | 49.02 | 2.72 | 54.9 | 83.5 | dup/MLL | MA | non-CR | 225 |
| AML37 | F | 63 | / | 3.72 | 3.3 | 212 | 74 | TP53 mutation | DAC | CR | 251 |
| AML38 | M | 27 | M3 | 9.3 | 5.67 | 318 | 20 | PML-RARA | ATRA, IDA | CR | 556 |
| AML39 | F | 62 | M4 | 132.73 | 2.51 | 76 | 79 | FLT3 | IA | non-CR | 54 |
| AML40 | F | 61 | / | 30.34 | 3.88 | 44.1 | 68 | ND | MA | non-CR | 4 |
| AML41 | F | 64 | M4 | 38.24 | 2.62 | 22 | 72.5 | BCR/ABL1, CBFβ/MYH11 | MA | CR | 81 |
| AML42 | F | 37 | M3 | 39.801 | 2.034 | 9.2 | 94 | PML/RARA, FLT3-ITD | ATRA, IDA | CR | 336 |
| AML43 | M | 26 | / | 280 | 2.69 | 17 | 74 | CBFb/MYH11 | MA | non-CR | 2 |
| AML44 | M | 18 | M1 | 99.4 | 2.3 | 44 | 59 | FLT3-ITD, KRAS, PTPN11, FAT1 | MA | non-CR | 209 |
| AML45 | F | 75 | / | 127.08 | 2.38 | 11 | 94 | FLT3-ITD，NPM1 | Ara-c | CR | 102 |
| AML46 | M | 66 | M5 | 72.9 | 1.52 | 48 | 28 | ND | Ara-c, Azacitidine | CR | 269 |
| AML47 | M | 35 | M2 | 6.44 | 4.4 | 47 | 63 | AML1-ETO, C-Kit | IA | CR | 155 |
| AML48 | M | 20 | M2 | 1.17 | 4.39 | 73 | 79 | AML1-ETO | MA | CR | 435 |
| AML49 | F | 54 | M5 | 138.5 | 3.63 | 61 | 90 | NPM1 | MA | non-CR | 11 |
| AML50 | M | 24 | / | 99.4 | 1.93 | 44 | 32 | ND | DA | CR | 1698 |
| AML51 | F | 47 | / | 134.53 | 2.33 | 48 | 60 | ND | IA | CR | 1798 |
| AML52 | M | 59 | / | 325.42 | 1.9 | 69 | 46 | ND | MA | non-CR | 17 |
| AML53 | F | 86 | M2 | 95.07 | 1.89 | 6.4 | 40 | ND | MA | non-CR | 11 |
| AML54 | M | 48 | M5 | 1.02 | 2 | 10 | 65 | FLT3-ITD, NPM1-Exon11, CEBPA, DNMT3A, TET2, ASXL1, TP53, WT1 | MA | CR | 807 |
| AML55 | M | 72 | M5 | 17.82 | 2.89 | 21 | 87.5 | ND | MA | non-CR | 7 |
| AML56 | M | 50 | M2 | 39.97 | 3.57 | 73 | 69 | AML1-ETO | IA | non-CR | 1222 |
| AML57 | M | 67 | M5 | 10 | 2.8 | 4 | 38 | ND | MA | non-CR | 45 |
| AML58 | M | 70 | M2 | 50.6 | 2.8 | 7.9 | 83 | MLL/AF10, WT1 | Azacitidine | non-CR | 60 |
| AML59 | M | 43 | M2 | 1.9 | 2.1 | 20 | 40 | K-RAS, DEK-CAN | DCAG | CR | 389 |
| AML60 | F | 43 | / | 27.5 | 1.3 | 31 | 25 | AML1-ETO, TET2 | IA | CR | 158 |
| AML61 | M | 40 | M2 | 4.1 | 2 | 40 | 26 | MLL-ELL | HSCT | CR | 377 |
| AML62 | F | 61 | / | 2.8 | 2.5 | 61 | 22 | TP53 | DCAG | CR | 416 |
| AML63 | F | 66 | M5 | 76.1 | 1.9 | 38.1 | 87 | MLL/AF9, EVI1 | IA | non-CR | 18 |
| AML64 | M | 64 | M4 | 4.8 | 2.2 | 51.6 | 30 | ND | IDA, Azacitidine | non-CR | 34 |
| AML65 | M | 82 | M4 | 22.8 | 1.7 | 81 | 63.5 | FLT3-ITD, NPM1, ASXL2, EZH2, TET2 | DAC | CR | 352 |
| AML66 | F | 37 | M5 | 4.9 | 3 | 632 | 63 | FLT3-ITD, CEBPA | IA | CR | 225 |
| AML67 | F | 62 | M5 | 132.7 | 2.5 | 76 | 79 | FLT3 | IA | non-CR | 54 |
| AML68 | F | 25 | / | 2.7 | 3.4 | 123 | 71 | ND | IA | CR | 345 |
| AML69 | M | 65 | / | 4.2 | 1.8 | 107 | 24 | NPM1, IDH1, DNMT3A | DA | non-CR | 711 |
| AML70 | M | 63 | M4 | 40.9 | 3.9 | 134 | 48 | FLT3, GATA2, NPM1, WT1 | D-CA | CR | / |
| AML71 | F | 52 | / | 1.1 | 1.5 | 30.2 | 24 | non-CRAS, TP53, STAG2 | IA | CR | 179 |
| AML72 | M | 88 | M5 | 45.9 | 3 | 132.5 | 61.5 | ND | MA | non-CR | 800 |
| AML73 | F | 56 | M4 | 1 | 1.9 | 26 | 29 | TET2, SF3B1 | DCAG | non-CR | 26 |
| AML74 | F | 47 | M2 | 5.70 | 3.5 | 251 | 85 | NPM1, non-CRAS, SRSF2, non-CRAS | D-IA | CR | 156 |
| AML75 | M | 77 | M5 | 30.8 | 2 | 86.8 | 30 | ND | Azacitidine, IA | non-CR | 264 |

Notes: WBC: white blood cell, RBC: red blood cell, PLT: platelet, F: female, M: male, ND: not detected, HSCT: hematopoietic stem cell transplantation, M0: minimally differentiated AML, M1: AML without maturation, M2: AML with maturation, M3: acute promyelocytic leukemia, M4: acute myelomonocytic leukemia, M5: acute monocytic leukemia, M6: pure erythroid leukemia, M7: Acute megakaryoblastic leukemia, CR: complete remission, non-CR: non complete remission, /: unknown, MA: mitoxantrone+cytarabine, ATRA: all-trans-retinoic acid, ATO: arsenic trioxide, DA: daunorubicin+ cytarabine, DCAG: decitabine+ aclarubicin + cytarabine+ recombinant granulocyte colony-stimulating factor, IA: idarubicin+ cytarabine, IDA: idarubicin, DCA: Decitabine, Ara-C:cytarabine, DAC：decitabine, MIT: mitoxantrone.
