# Supplementary material for: High TRGV 9 Subfamily Expression Marks an Improved Overall Survival in Patients With Acute Myeloid Leukemia
Source: Front Immunol. 2022 Feb 10;13:823352. doi: 10.3389/fimmu.2022.823352 (PMC8866455; doi:10.3389/fimmu.2022.823352)
Supplement: Supplementary file 2 [file Table_2.doc]

| Primer | Sequence |
| --- | --- |
| VG1 | 5’-TACCTACACCAGGAGGGGAAG-3’ |
| VG2 | 5’-GGCACTGTCAGAAAGGAATC-3’ |
| VG3 | 5’-TCGACGCAGCATGGGTAAGAC-3’ |
| Cγ | 5’- GTTGCTCTTCTTTTCTTGCC-3’ |
| Cγ-FAM | 5’-FAM-CATCTGCATCAAGTTGTTTATC -3’ |
| *β*2M-for | 5’-TACACTGAATTCACCCCCAC |
| *β*2M-back | 5’-CATCCAATCCAAATGCGGCA |

**Supplementary Table 2 Primers for RT-qPCR**
